# An Assessment and Extension of the Mechanism-Based Approach to the Identification of Age-Period-Cohort Models

**DOI:** 10.1007/s13524-017-0562-6

**Published:** 2017-03-09

**Authors:** Maarten J. Bijlsma, Rhian M. Daniel, Fanny Janssen, Bianca L. De Stavola

**Affiliations:** 1 Unit PharmacoEpidemiology & PharmacoEconomics (PE2), Department of Pharmacy, University of Groningen, A. Deusinglaan 1, Groningen, 9713 AV The Netherlands; 2 Max Planck Institute for Demographic Research, Rostock, Germany; 3 Centre for Statistical Methodology and Department of Medical Statistics, London School of Hygiene and Tropical Medicine, London, UK; 4 Population Research Centre (PRC), Faculty of Spatial Sciences, University of Groningen, Groningen, The Netherlands; 5 Netherlands Interdisciplinary Demographic Institute, University of Groningen, The Hague, The Netherlands

**Keywords:** Age-period-cohort analysis, Identification, Causal inference, Mechanisms, Front-door criterion

## Abstract

Many methods have been proposed to solve the age-period-cohort (APC) linear identification problem, but most are not theoretically informed and may lead to biased estimators of APC effects. One exception is the mechanism-based approach recently proposed and based on Pearl’s front-door criterion; this approach ensures consistent APC effect estimators in the presence of a complete set of intermediate variables between one of age, period, cohort, and the outcome of interest, as long as the assumed parametric models for all the relevant causal pathways are correct. Through a simulation study mimicking APC data on cardiovascular mortality, we demonstrate possible pitfalls that users of the mechanism-based approach may encounter under realistic conditions: namely, when (1) the set of available intermediate variables is incomplete, (2) intermediate variables are affected by two or more of the APC variables (while this feature is not acknowledged in the analysis), and (3) unaccounted confounding is present between intermediate variables and the outcome. Furthermore, we show how the mechanism-based approach can be extended beyond the originally proposed linear and probit regression models to incorporate all generalized linear models, as well as nonlinearities in the predictors, using Monte Carlo simulation. Based on the observed biases resulting from departures from underlying assumptions, we formulate guidelines for the application of the mechanism-based approach (extended or not).

**Electronic supplementary material:**

The online version of this article (doi:10.1007/s13524-017-0562-6) contains supplementary material, which is available to authorized users.

## Introduction

Demographers, epidemiologists, sociologists, and others have attempted to break down outcomes of interest into constituent effects caused by, or associated with, age, calendar time, and time of birth—an approach known as *age-period-cohort* (APC) *analysis*. *Age effects* refer to changes in the outcome as the age of individuals in the study population progresses. For example, as individuals age, cardiovascular function declines, and hence older individuals tend to have worse cardiovascular health than younger individuals. *Period effects* refer to changes that occur in an outcome as calendar time progresses. They can represent sudden changes or temporary changes in an outcome, such as spikes in death rates due to war or famine, but may also represent gradual changes such as those produced by the accumulation of minor improvements in public health infrastructure over time that influence mortality rates in all age groups. Finally, *birth cohort effects* represent differences between generations that are not attributable to differences in age or calendar time. Conceptually, they commonly represent the effects of shared formative experiences of individuals in a birth cohort, either in utero or during other critical phases in the life course (Ben-Shlomo and Kuh 2002). The effect of these formative years would then remain largely constant in that cohort throughout the remaining life course and are therefore independent of age and calendar time. For example, the cohort that was in utero during the Dutch Hunger Winter in 1944–1945 had worse health even later in life (Ekamper et al. 2014) compared with other cohorts. Furthermore, birth cohort has been found to be strongly tied to smoking behavior in various Western countries (Preston and Wang 2006; Verlato et al. 2006).

Unfortunately, decomposing an outcome (*Y*) into the separate effects of age (*A*), period (*P*), and cohort (*C*) using, for example, a linear regression model (e.g., E(*Y*|*A*,*P*,*C*) = η + α *· A* + β *· P* + θ · *C*) imposes an identification problem. Because *A* = *P* – *C*, these three variables are linearly dependent, and consequently any linear model involving these three variables cannot have a unique solution. To circumvent this, researchers have introduced various techniques that constrain the model specification (e.g., Clayton and Schifflers 1987; Held and Riebler 2012; Holford 2006; Yang et al. 2008) so that a solution can be found. However, the technical constraints that have been proposed are arbitrary and do not lead to meaningful measures of effect (Bell and Jones 2014; Luo 2013). Estimation of the parameters may be unbiased but only under the constraints that have been imposed, and hence the estimates do not reflect the true effects of age, period, and cohort that we seek (Luo 2013). Luo and Fienberg both argued in favor of a paradigm shift in a recent discussion in *Demography* (Fienberg 2013; Luo 2013): APC analysis, they argued, needs to become more theoretically informed. Simply fitting a regression model to an outcome given age, period, and cohort, without any forethought or theoretical reasoning, cannot result in meaningful effect estimates for these variables.

Few authors have explicated what they mean by *true* or *meaningful* effect estimates. Viewed from one perspective, because the relationship *A* = *P* – *C* always holds, a regression model with age, period, and cohort as covariates truly has infinitely many solutions, and thus there is no problem to be solved. However, because those who write about this problem talk of one special solution of those infinitely many solutions that is correct/true/valid/meaningful, it must be that they are (albeit implicitly) thinking of a hypothetical world, different from the actual world, in which age, period, and cohort can be manipulated such that the identity *A* = *P* – *C* is broken.

More formally, one could take an explicitly causal perspective using potential outcomes with age, period, and cohort as independent exposures. Let *Y*(*a,p,c*) be the potential outcome that would occur if *A* were set to *a*, *P* were set to *p*, and *C* were set to *c*, without necessarily abiding by the relationship *a* = *p* – *c* (Rubin 1974). Then the causal model,

1 $$ E\left( Y\left( a, p, c\right)\right)={\upeta}^{\ast }+{\upalpha}^{\ast}\cdot a+{\upbeta}^{\ast}\cdot p+{\uptheta}^{\ast}\cdot c, $$

has one solution, and this is presumably the true solution to which the various authors on this topic refer.

In fact, imagining a hypothetical world in which time can be manipulated is difficult enough. Contemplating one in which three different aspects of time—namely, age, period, and cohort—can be independently manipulated requires an even wilder imagination and is therefore unlikely to be truly of interest. More realistically, we can view Eq. (1) as being shorthand for

2 $$ E\left( Y\left({c}_a,{c}_p,{c}_c\right)\right)={\upeta}^{\prime }+{\upalpha}^{\prime}\cdot {c}_a+{\upbeta}^{\prime}\cdot {c}_p+{\uptheta}^{\prime}\cdot {c}_c, $$

where *c*_*a*_, *c*_*p*_, and *c*_*c*_ are the set of all immediate *consequences* of age, period, and cohort, respectively. Thus, if being born in a particular cohort meant being born during a famine, it is this famine that we imagine we could manipulate—say, “prevent”—rather than the cohort of birth itself. But because we may not have all these consequences at our disposal, Eq. (2) is replaced (as a shorthand) by Eq. (1).

Given this reframing of the model of interest as a causal model, it makes sense to consider methods from causal inference (Pearl 2000) to analyze data from APC studies. Undertaken by Winship and Harding (2008), this was dubbed the “mechanism-based approach.” In particular, their approach uses Pearl’s front-door criterion to identify the APC causal parameters α*, β*, and θ* (Pearl 2000). In short, the mechanism-based approach uses intermediate variables on the path between one of the three APC variables and the outcome in order to estimate (1) the effect of one of these three variables on the outcome indirectly, and (2) the effect of the remaining two APC variables directly (with the method generalizable to modeling intermediate variables for two of the three APC variables). The approach naturally leads to drawing a directed acyclic graph (DAG) (Glymour 2006; VanderWeele et al. 2008) depicting the assumed relationships among *A*, *P*, *C*, the intermediate variables being considered, and the outcome. It thus motivates researchers to be explicit about their substantive assumptions.

The method requires that a complete set of intermediate variables can be found for at least one of the three APC variables. By a *complete* set of intermediate variables for *A*, for example, we mean a set of variables *M*_1_, *M*_2_, . . . ,*M*_*K*_ that are affected by age and which themselves affect the outcome *Y* in such a way that *all* the effect of *A* on *Y* is via this set of intermediate variables.

However, in a realistic setting, finding a complete set of intermediate variables even for just one of the three APC variables is unlikely. Also, the partial set of intermediate variables that may be available could be dependent on more than one APC variable. Furthermore, there may be variables that affect both the intermediate variable(s) and the outcome. All these settings (if they cannot somehow be accounted for) threaten the mechanism-based approach with bias, and one of the aims of this article is to demonstrate these potential sources of bias and their magnitude in these realistic scenarios.

Another challenge also arises: the mechanism-based approach has been developed for use in linear and probit regression models for *Y*, and in linear and probit regression models for the intermediate variables *M*_1_, *M*_2_, . . . ,*M*_*K*_. Although some analytical solutions (e.g., Winship and Mare 1983) could be adopted to extend this approach to using logistic regression models for outcome and/or mediators, they are complex to implement. Moreover, only approximate methods are available to deal with settings where the variables included in the outcome model interact or have some other nonlinear effects, even when *Y*, *M*_1_, *M*_2_, . . . ,*M*_*K*_ are all continuous and modeled using linear models (Jiang and VanderWeele 2015; Preacher and Hayes 2008; VanderWeele 2015).

In this article, in order to illustrate possible pitfalls one may encounter using mechanism-based APC models, we assess their performance under realistic settings: namely when (1) only a partial set of mediators is available, (2) some of the intermediate variables are affected by two or more of the APC variables (a feature that is not acknowledged in the analysis), and (3) unmeasured confounding affects the intermediate variables and the outcome. Furthermore, we extend the mechanism-based approach to settings with any fully parametric model for the outcome and intermediate variables by approximating the estimation of the APC parameters using Monte Carlo simulation. R code demonstrating the mechanism-based approach, and its extension, is available in Online Resource 1.

## Methods

### The Mechanism-Based Approach

The mechanism-based approach exploits the fact that age, period, and cohort affect the outcome through intermediate variables (Winship and Harding 2008). The key idea is that while age, period, and cohort are deterministically related, the intermediate variables along the paths from these to the outcome (hereafter, *mediators*) will be affected by other (APC-independent) causes and hence can be used (if measured) to circumvent the identification problem. We now discuss this in more detail.

**Fig. 1:**
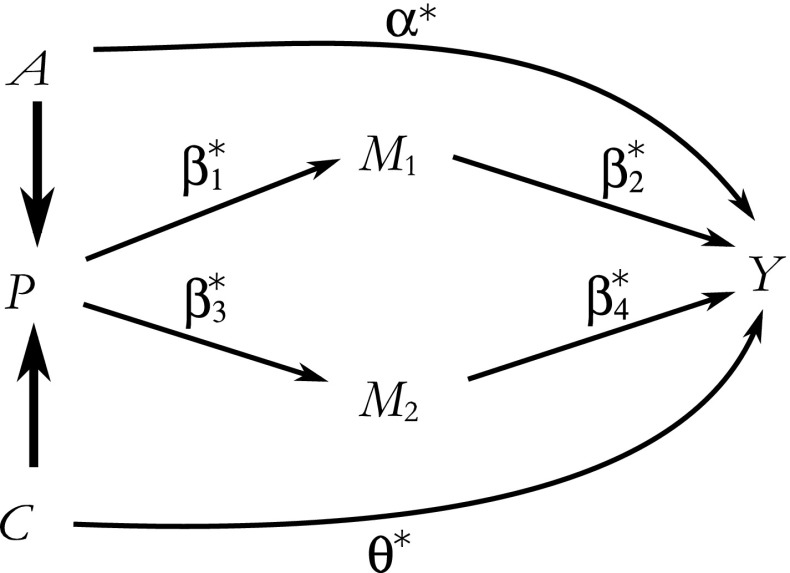
Causal directed acyclic graph, showing the age effect (α*), cohort effect (θ*), and the period effect (the β*s). The bold arrows represent deterministic relationships, and the nonbold arrows represent stochastic relationships

Consider for simplicity the setting depicted in Fig. 1, which shows a causal directed acyclic graph (DAG). Causal DAGs are formal graphical representations of the assumed causal relationships between the variables under study (Glymour 2006). Here, the number of mediators *K* is equal to 2, and the mediators *M*_1_ and *M*_2_ being considered lie on causal pathways from *P* to *Y*. Note that there is no arrow from *P* to *Y* in the DAG, representing the assumption that all the effect of *P* on *Y* is via *M*_1_ and *M*_2_. Also note that there is no arrow from either *A* or *C* to the mediators, nor shared common causes of the mediators and any other variables in the DAG. Finally, the two paths from *P* to *Y* are separate in the sense that *M*_1_ and *M*_2_ do not affect each other, nor does any variable along either path affect a variable on the other path or share any common causes: *M*_1_ and *M*_2_ are assumed to be conditionally independent given *P*. These strong structural assumptions concerning the roles of *M*_1_ and *M*_2_ (some of which can be relaxed, which we discuss in Online Resource 2) allow the identification of the APC effects in a two-stage procedure.

In the first step, separate models for each mediator on *P* are fitted. In the second step, a model for the outcome *Y* on *A*, *C*, *M*_1_, and *M*_2_ is fitted. If Fig. 1 is correct, and the outcome and the mediators are continuous variables and modeled using linear regression, or the outcome and mediators are binary and modeled using probit regression models, and none of these models in truth includes product terms or other nonlinearities, then the effect of *A* and *C* on *Y* (α* and θ*) is equal to their regression coefficients in the outcome (second-step) model, while the effect of *P* on *Y* (β*) is equal to the sum of the effects along the two pathways involving *M*_1_ and *M*_2_. The effect along a pathway is equal to the product of two regression coefficients; for the *P* – *M*_1_ – *Y* pathway, it is the product of the coefficient for *P* in the (first-step) regression of *M*_1_ on *P* and the coefficient for *M*_1_ in the (second-step) regression of *Y* (that also includes *M*_2_, *A*, and *C* as covariates). Similar calculations apply to the *P* – *M*_2_ – *Y* pathway, and the effects along the two pathways are then summed to obtain the effect of *P* on *Y*. These calculations are an application of the path-tracing rules that are widely used in structural equation modeling (Mulaik 2009; Wright 1934), with standard errors for the estimated effect of *P* estimated using the delta method (in simple settings) or, more generally, the bootstrap (MacKinnon et al. 2004). See Online Resource 2 for an applied example of the path-tracing rule.

### Complications

In a real-life setting, a number of situations may occur that make mechanism-based estimation of APC effects less straightforward. First, if a complete set of mediators is not available for the selected APC variable(s), then the effect estimators of the three APC variables described earlier will be biased for α*, β*, and θ* because the required assumption that (at least) one of the three APC variables is fully mediated by a set of measured mediators would not be met. Second, a variable that we believe to be a mediator for one of the three APC variables may actually be a mediator for more than one. In this case, the regression coefficient for the APC variable that we did not believe to be mediated by any of the mediators for *P*, *M*_1_, *M*_2_, . . . ,*M*_*K*_ will capture only the component of its effect that is not mediated. Third, the relationship between mediators and outcome may be confounded: that is, a variable (either measured or unmeasured) may have a causal effect on both the mediator(s) and the outcome. If this confounding is not controlled for in the outcome model, the effect of the mediator on the outcome will be estimated with bias, and consequently also the effect of the APC variable that is assumed to be mediated by it. Finally, the outcome and/or the mediators may not be of a type that can be modeled by linear or probit regression, or even if they can, when the models require product terms or other nonlinearities. In this situation, the path-tracing rule needed to derive the causal effect of the mediated APC variable cannot be used (Mulaik 2009).

### Simulation: Approach

We assess the mechanism-based approach through simulations. In our simulations, we attempt to re-create a realistic setting in which APC analyses are performed: namely, the study of cardiovascular mortality. However, to demonstrate particular pitfalls, we isolate sources of bias in the APC effect estimates and therefore simplify this real-world setting into three scenarios.

In each scenario, the study population and outcome are the same. The individuals in the study population are generated to be aged 40–95 years during the calendar years 1990–2015, and hence the whole data set comprises birth cohorts from birth years 1896–1975. Age and period for each record are generated according to uniform distributions but are then categorized into five-year groups (for *A* and *P*) and cohort is dependent on these categories (*C* = *P* – *A*). The outcome is mortality due to cardiovascular disease (CVD), coded as 1 (death due to CVD) or 0 (alive or dead from other causes). It was generated in all scenarios according to either a probit or logistic regression model, with the probability of CVD death generated as a function of age, cohort, and the period mediators. Age is set to account for 70 % of the effect of the APC variables on cardiovascular mortality; period (via its mediators) for 20 %; and birth cohort for 10 %. We believe that these percentages approximately correspond to realistic effects of age, period, and cohort in the period 1990–2015 in Western countries. The difference in incidence of CVD death between individuals aged 40 and those aged 95 is very large, whereas the difference in incidence of CVD death in these age groups between the year 1990 and 2015 is much smaller (Peeters et al. 2011). Because of the linear dependency phenomenon, it is unknown what part of these differences is truly attributable to each dimension.

### Simulation: Mediators and Confounders

We simulate settings where *P* is the variable that has measured mediating variables. Results, however, easily generalize to the alternative scenarios where *A* or *C* play this role, with due numerical differences given their unequal assumed strength of effects. Four mediators on the path from *P* to *Y* are included in the simulation study: *body mass index* (*BMI*), *smoking*, *statin therapy*, and *unmeasured*. We choose the first three variables because they are commonly described variables that are believed to affect CVD mortality; many other variables that are also believed to affect CVD mortality (e.g., Blackmore and Ozanne 2015; Capewell et al. 2000) are represented by the *unmeasured* variable. Together, these four variables account for the entire period effect on the outcome. The *unmeasured* and *BMI* variables are continuous, whereas *smoking* and *statin therapy* are binary. We set each of the measured mediators to account for ~20 % of the period effect on cardiovascular mortality; we set the *unmeasured* mediator to account for ~40 %.

Table 1 shows the direction of the effects of period on the mediators as well as each mediator on the outcome. The effect of period on the *unmeasured* mediators is linear, and its effect on the measured mediators is nonlinear but monotonically increasing or decreasing. Initially, these variables are made to act as mediators only on the path between *P* and CVD mortality; however, in some scenarios, they are also affected by *A* or *C*, in which case the total effects of these latter variables changes. In the scenario that includes confounding (see the section, Simulation: Scenarios and Variants), the confounder is presence or absence of a particular gene, randomly assigned to be present in 50 % of individuals and set to have a positive effect on both the mediators and the outcome (Sabol et al. 1999; Smith and Newton-Cheh 2015).

**Table 1:** Direction of effect of period on the mediators, mediators on cardiovascular mortality, and the direction along the entire path of period on cardiovascular mortality

	Direction of Effect		
Mediator	Period on Mediator	Mediator on Outcome	Period on Outcome
Unmeasured	Negative	Positive	Negative
BMI	Positive	Positive	Positive
Smoking	Negative	Positive	Negative
Statin	Positive	Negative	Negative

### Simulation: Scenarios and Variants

**Fig. 2:**
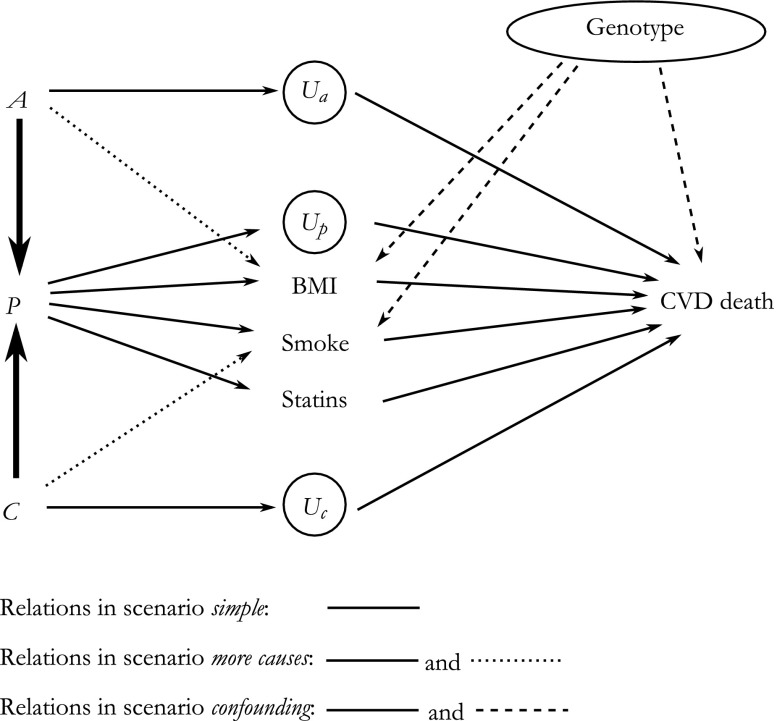
Causal directed acyclic graph of the three scenarios investigated by simulation. Bold arrows between age (*A*), period (*P*), and cohort (*C*) represent a deterministic relationship, whereas the remaining arrows represent stochastic causal relationships. Circled variables represent variables that are omitted from the estimation model (in some simulation set-ups)

In all simulations, we generate data for 100,000 individuals, each measured once. We simulate three scenarios, with each scenario simulated 1,000 times. In the data-generating process for the first scenario (*simple*), *A* and *C* have a direct effect on *Y*, and only the effect of *P* is mediated. In the second scenario (*more causes*), *A* has an additional (negative) effect on the mediator BMI, which amounts to roughly 30 % of the total age effect, and *C* has an additional (positive) effect on smoking, which amounts to roughly 30 % of the total cohort effect. Finally, in the third scenario (*confounding*), genotype confounds the relationship between BMI and *Y* and between smoking and *Y* (Fig. 2): genotype has a positive effect on both BMI and smoking, and has a positive effect on CVD mortality. Genotype accounts for roughly 33 % of the association between age and CVD death and 35 % of association between cohort and CVD death.

Each scenario has two variants. In the first variant, we generate *Y* and the binary mediators using probit regression models; in the second variant, logistic regression models are used instead. In both variants, linear regression models are used to generate continuous variables. Because probit and logistic regression models transform parameters into probabilities in a different way, the probabilities of cardiovascular mortality somewhat differ between the two variants. We varied the value for the intercept in each variant so that the age-specific probabilities of cardiovascular mortality were similar to those found in high-income countries. However, because of these differences in transformation, the extent of the bias found with the variants is not directly comparable.

Finally, to demonstrate how the unequally distributed strength of age, period, and cohort affect the bias, we perform two sets of simulations in which we vary these strengths. In the first set, we vary the size of the period effect from 0 % to 100 % in 20 % increments, while correspondingly reducing the size of the age effect and keeping the size of the cohort dimension constant at 10 % (excluding the last increment, where cohort is necessarily set to 0 %). The second set is identical, but then the cohort effect size is varied and period kept constant at 20 %. In both sets, bias is generated by removing the first mediator (*unmeasured*) from the estimation model in the *simple* scenario. These simulations were done with probit, logistic, and linear regression variants. The logistic and linear regression variants, plus a third set of simulations in which the age effect size is varied, are described in Online Resource 2.

### Simulation: Estimation

Estimation of the APC effects according to the mechanism-based approach consists of fitting separate regression models for CVD death as function of *A*, *C*, and the *P* mediators and of each of the mediators as functions of *P*. In all estimation models, *A*, *P*, and *C* are treated as categorical variables through dummy coding (10 dummy variables for age, 4 dummy variables for period, and 14 dummy variables for cohort because cohort categories were forced to overlap in order to maintain the linear identity, *C* = *P* – *A*). By generating cohort in this way, we maintain the linear dependency among age, period, and cohort and therefore follow the equal interval width definition (Luo and Hodges 2016).

Following how we generated the variables in each variant, in the probit variant, we use probit regression models for CVD death and logistic regression models for the logistic variant. The effects of *P* (and *A* and *C* when appropriate) on continuous mediators are estimated using linear regression and for binary mediators using logistic or probit regression models. In all three scenarios, we first perform our estimation under entirely correct assumptions. That is, in the second scenario, we also model the additional paths from *A* and *C* to their mediators (as described in Online Resource 2); and in the third scenario, we also control for the confounder. Moreover, in all scenarios, the parametric forms used to fit these models are the same as those used to generate the data. Then, to investigate the effect of incorrect assumptions, in the second scenario, we omit to model the path from *A* and *C* to the mediators; and in the third scenario, we do not control for confounding. Additionally, to explore the effect of including an incomplete set of mediators, in all three scenarios, we first remove the *unmeasured* mediators from the estimation model, then *BMI*, followed by *smoking*; finally, we remove all period mediators (i.e., fit an age-cohort model). For completeness, we also report the results of adopting a more traditional APC approach in Online Resource 2.

Results obtained from each scenario, variant, and model specification are summarized as means of each parameter’s estimates over the 1,000 simulations, which we compare with the estimated values obtained from the correctly specified models to estimate the bias. Because of the very large sample size, the estimates obtained from the correct models can be interpreted as the true values.

### Extending the Mechanism-Based Approach Through Monte Carlo Integration

When a model with a general nonlinear link function is used for a mediator or for the outcome (or both)—for example, Poisson or logistic regression—or if the models include product terms or other nonlinearities, the path-tracing method cannot be used (Mulaik 2009). A different approach is then required.

The basic intuition of our approach is as follows. First, similar to the traditional approach, we estimate individual relations among age, period, cohort, and mediators, and then between mediators and outcome (Step 1 and Step 2, respectively). The difference now is that the statistical models used for these steps are allowed to have nonlinear functional forms. However, this enhanced flexibility comes at a price: the traditional multiplication of coefficients along pathways is no longer possible. Therefore, Monte Carlo integration is used instead (Robert and Casella 2004); the coefficients from Steps 1 and 2 are used in Steps 3 and 4 to generate a new data set that does not suffer from APC linear dependency (Pearl’s front-door criterion makes this possible) but that reflects the original data structure. In Step 5, an APC model is then fitted to this newly created data set to provide estimates of the effects of age, period, and cohort. Our approach is, in many ways, analogous to the Monte Carlo estimation of the parametric g-formula (Hernán and Robins 2013; Keil et al. 2014). A similar approach has also been suggested in mediation analysis (VanderWeele 2015).

We treat age, period, and cohort as continuous variables to simplify the presentation. However, in our simulations, we model age, period, and cohort as categorical variables. Furthermore, we describe here only the case where there is one mediator for period and where the mediator has only one cause. See Online Resource 2 for a description of a more general setting. We proceed in six steps.

Step 1. **Mediator estimation:** Fit a model for the mediator. If it is continuous, we can use linear regression; for example,

$$ M={\upgamma}_0+{\upgamma}_1 \cdot P+\nu, $$

where we assume $\nu \sim N\Big(0,{\upsigma}_M^2$). Note that the assumption on the distribution of the error terms in the linear regression model is nontrivial if there are nonlinearities involving *M* in the model for *Y*. If instead a mediator is binary, we can use logistic regression; for example,

logit{*E*(*M*| *P*)} = γ_0_ + γ_1_ ⋅ *P*.

Let $\left({\widehat{\upgamma}}_0,{\widehat{\upgamma}}_1\right)$ be the estimates of (γ_0_, γ_1_) from the appropriate model. If the mediator is continuous, also save the estimate of the error variance, ${\widehat{\upsigma}}_M^2$. These estimates will be used in Step 3.

Step 2. **Outcome estimation:** Fit a model for the outcome. If the outcome is continuous, fit a model using linear regression; for example,

$$ Y={\updelta}_0 + {\updelta}_1\cdotp A+{\updelta}_2\cdotp C+{\updelta}_3\cdotp M+\upxi, $$

where $\upxi \sim N\Big(0,{\upsigma}_Y^2$). If the outcome is binary, we use logistic regression; for example,

$$ \mathrm{logit}\left\{ E\left( Y| A, M, C\right)\right\}={\updelta}_0 + {\updelta}_1\cdotp A+{\delta}_2\cdotp C+{\updelta}_3\cdotp M. $$

Let $\left({\widehat{\updelta}}_0,{\widehat{\updelta}}_1,{\widehat{\updelta}}_2,{\widehat{\updelta}}_3\right)$ be the estimates of (δ_0_, δ_1_, δ_2_, δ_3_) from the appropriate model. If the outcome is continuous, also save the estimate of the error variance, ${\widehat{\upsigma}}_Y^2$. These estimates will be used in Step 4.

Step 3. **Mediator simulation:** For each of a range of period values, *p*, simulate the mediator $\overset{\sim }{M}\left(\overset{\sim }{p}\right)$. The values $\overset{\sim }{p}$ could be randomly generated—for example, using a discrete uniform distribution—but their range should be equal to the range empirically observed in the data that were used for estimation. For example, if we have data ranging from 1990 to 2015, that would be the range of values for $\overset{\sim }{p}$ that we use. If instead we categorize this into five-year periods (1990–1994, 1995–1999, and so on), then we generate values of $\overset{\sim }{p}$ corresponding to these categories. If a mediator is continuous, use the estimates of the linear regression model in Step 1 to simulate

$$ \tilde{M}\left(\tilde{p}\right)={\widehat{\upgamma}}_0+{\widehat{\upgamma}}_1 \cdot \tilde{p}+\widehat{\nu}, $$

where $\widehat{\nu}$ is randomly drawn from $N\Big(0,{\widehat{\upsigma}}_M^2$). If instead the mediator is binary, use the estimates from the logistic regression model in Step 1 to simulate $\overset{\sim }{M}$ *(* $\overset{\sim }{p}\Big)$ from a Bernoulli distribution with mean

$$ {\widehat{\upmu}}_{m,\tilde{p}}=\frac{ \exp \left({\widehat{\upgamma}}_0+{\widehat{\upgamma}}_1 \cdot \tilde{p}\right)\ }{1+ \exp \left({\widehat{\upgamma}}_0+{\widehat{\upgamma}}_1 \cdot \tilde{p}\right)\ }. $$

The number of $\overset{\sim }{M}\left(\overset{\sim }{p}\right)$ values to be simulated need not be equal to the number of observations in the data as long as the entire empirical range is covered—but the more values we simulate, the less our final estimates will be affected by Monte Carlo error. The values of $\overset{\sim }{M}\left(\overset{\sim }{p}\right)$ will be used in Step 4.

Step 4. **Outcome simulation:** For each of a range of age, period, and cohort values (*a*, *p*, *c*), simulate the potential outcome $\overset{\sim }{Y}\left(\overset{\sim }{a},\overset{\sim }{p},\overset{\sim }{c}\right)$. Because $\overset{\sim }{p}$ is already generated in Step 3, the values $\overset{\sim }{p}$ can be reused instead of regenerated. As previously, the range of these values should be equal to the range empirically observed in age, period, and cohort in the data, respectively. However, we choose $\overset{\sim }{a}$, $\overset{\sim }{p}$, and $\overset{\sim }{c}$ independently—that is, the identity $\overset{\sim }{a}=\overset{\sim }{p} - \overset{\sim }{c}$ should not hold. If the outcome is continuous, then using the linear regression estimates of Step 2 and the simulated mediator values of Step 3 simulate the following:

$$ \tilde{Y}\left(\tilde{a},\tilde{p},\tilde{c}\right)={\widehat{\updelta}}_0+{\widehat{\updelta}}_1 \cdot \tilde{a}+{\widehat{\updelta}}_2 \cdot \tilde{c}+{\widehat{\updelta}}_3 \cdot \tilde{M}\left(\tilde{p}\right)+\widehat{\upxi}, $$

where $\widehat{\upxi}$ is randomly drawn from $N\Big(0,{\widehat{\upsigma}}_Y^2$), and $\overset{\sim }{M}\left(\overset{\sim }{p}\right)$ is as generated in Step 3. If instead the outcome is binary, use the estimates from the logistic regression model in Step 2 and the simulated mediator values of Step 3 to simulate $\overset{\sim }{Y}\left(\overset{\sim }{a},\overset{\sim }{p},\overset{\sim }{c}\right)$ from a Bernoulli distribution with mean calculated as follows:

$$ {\widehat{\upmu}}_{y,\tilde{a},\tilde{p},\tilde{c}}=\frac{ \exp \left({\widehat{\updelta}}_0+{\widehat{\updelta}}_1 \cdot \tilde{a}+{\widehat{\updelta}}_2 \cdot \tilde{c}+{\widehat{\updelta}}_3 \cdot \tilde{M}\left(\tilde{p}\right)\right)\ }{1+ \exp \left({\widehat{\updelta}}_0+{\widehat{\updelta}}_1 \cdot \tilde{a}+{\widehat{\updelta}}_2 \cdot \tilde{c}+{\widehat{\updelta}}_3 \cdot \tilde{M}\left(\tilde{p}\right)\right)\ }. $$

Step 5. **APC effect estimation:** Estimate age, period, and cohort effects using the simulated values $\overset{\sim }{Y}\left(\overset{\sim }{a},\overset{\sim }{p},\overset{\sim }{c}\right)$. If $\overset{\sim }{Y}\left(\overset{\sim }{a},\overset{\sim }{p},\overset{\sim }{c}\right)$ was generated as a continuous variable, use linear regression with $\overset{\sim }{a}$, $\overset{\sim }{p}$, and $\overset{\sim }{c}$ as covariates. If instead $\overset{\sim }{Y}\left(\overset{\sim }{a},\overset{\sim }{p},\overset{\sim }{c}\right)$ was generated as a binary variable, use logistic regression with $\overset{\sim }{a}$ *,* $\overset{\sim }{p}$, and $\overset{\sim }{c}$ as covariates. These models will be identifiable because $\overset{\sim }{a}$, $\overset{\sim }{p}$, and $\overset{\sim }{c}$ should have been chosen independently. The estimated parameters can be interpreted as the age, period, and cohort effects from the causal model (Eq. (1)).
Step 6. **Standard error estimation:** Use the nonparametric bootstrap to estimate the standard errors for the parameter estimates (Efron and Tibshirani 1994). This step consists of resampling with replacement from the original data, a data set of equal size, and then repeating Steps 1–5, and saving the parameter estimates of the effects of $\overset{\sim }{a}$, $\overset{\sim }{p}$, and $\overset{\sim }{c}$ at the end of Step 5. The standard deviations of the distributions of the $\overset{\sim }{a}$, $\overset{\sim }{p}$, and $\overset{\sim }{c}$ effect estimates can be used as estimates of the standard errors; alternatively, for example, the empirical 2.5 % and 97.5 % quantiles of these distributions can be used to derive 95 % confidence intervals directly (with improvements such as bias-corrected and accelerated intervals to be recommended).

Online Resource 1 includes R code demonstrating the application of this technique. In linear settings, this technique results in findings identical to the traditional path-tracing method, as long as a sufficiently large number of Monte Carlo simulations are carried out.

## Linear Dependency and Expected Bias

Although age, period, and cohort could be pairwise-independent, the three variables together have a deterministic relationship: *A* = *P* – *C*. This linear dependency will determine the (direction of the) bias when the mechanism-based approach is used and not all the mediators are included in the estimation model. We demonstrate this here.

In a linear setting, the causal model described in Eq. (1) could be expressed as

$$ Y\left( a, p, c\right)={\upeta}^{\ast }+{\upalpha}^{\ast } \cdot a+{\upbeta}^{\ast } \cdot p+{\uptheta}^{\ast } \cdot c+{\upvarepsilon}^{\ast }, $$

where α*, β*, and θ* represent the effects of age, period, and cohort; η*, the intercept; and ε*, the mean-zero error terms. This model corresponds to an (unidentified) associational model:

3 $$ Y=\upeta +\upalpha \cdot A+\upbeta \cdot P+\uptheta \cdot C+\upvarepsilon . $$

Because *P* = *A* + *C*, we can rewrite Eq. (3) as

4 $$ Y=\upeta +\upalpha \cdot A+\upbeta \cdot \left( A+ C\right)+\uptheta \cdot C+\upvarepsilon =\upeta +\left(\upalpha +\upbeta \right) \cdot A+\left(\uptheta +\upbeta \right) \cdot C+\upvarepsilon . $$

Eq. (4) shows that if we fit an age-cohort model (omitting period) and interpret the coefficients of *A* and *C* as representing the age and cohort effect, respectively, the period effect is attributed to age and cohort in equal parts, thereby biasing the presumed age and cohort effects in the direction of the period effect. In the same way, because *A* = *P* – *C*, and *C* = *P* – *A*, we have

5 $$ Y=\upeta +\left(\upbeta +\upalpha \right) \cdot P+\left(\uptheta -\upalpha \right) \cdot C+\upvarepsilon $$

6 $$ Y=\upeta +\left(\upalpha -\uptheta \right) \cdot A+\left(\upbeta +\uptheta \right) \cdot P+\upvarepsilon $$

when fitting period-cohort and age-period models, respectively. As Eqs. (5) and (6) show, in the period-cohort model, the period parameter is biased in the direction of the age effect, while the cohort parameter is biased in the opposite direction. Similarly, in the age-period model, the period parameter is biased in the direction of the cohort effect, while the age parameter is biased in the opposite direction. Of course, in all three models, the effect of the omitted variable would also be biased (unless its effect is null) given that its effect estimate is effectively set to 0.

**Fig. 3:**
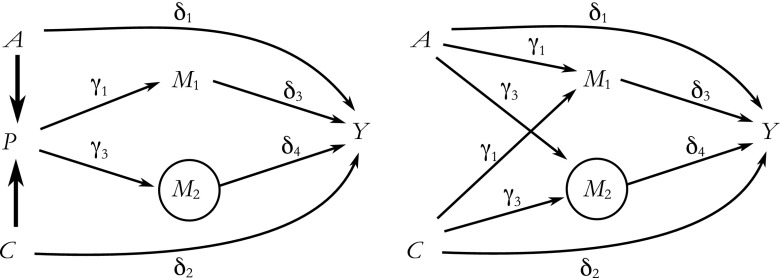
Causal directed acyclic graph when the age effect (δ_1_) and cohort effect (δ_2_) are estimated directly, and the period effect is estimated using mediators as per Pearl’s front-door criterion, while one period mediator is unmeasured. Left: effect estimates if *M*_*2*_ is measured, and *P* is included in the estimation process. Right: relationships that form due to linear dependency when period is excluded from estimation

The same logic applies when we use mediators to estimate the effects of age, period, or cohort while estimating the other two effects directly. Consider our earlier example in which we use two mediators on the period path but estimate the effects of age and cohort directly (Fig. 1). However, this time, we omit the variable *M*_*2*_ from our estimation model, perhaps because it was not measured (Fig. 3, left). When fitting a model for the outcome (*Y*) using *A*, *C*, and the measured period mediator (*M*_1_) as explanatory variables, there will be an association between *A* and the period mediators and between *C* and the period mediators because of the aforementioned linear dependency (Fig. 3, right). Because the model for *Y* includes *M*_1_ (i.e., we condition on *M*_1_), the paths from *A* or *C* to *Y* via *M*_1_ are blocked. However, because we do not condition on the unmeasured mediator (*M*_2_), the pathways from *A* and *C* to *Y* via *M*_2_ are not blocked and hence their regression parameters will be biased by the contribution of the additional paths from, respectively, *A* and *C* via *M*_2_ (i.e., δ_4_ ∙ γ_3_ for both).

## Results

### Scenario 1: *Simple*

**Fig. 4:**
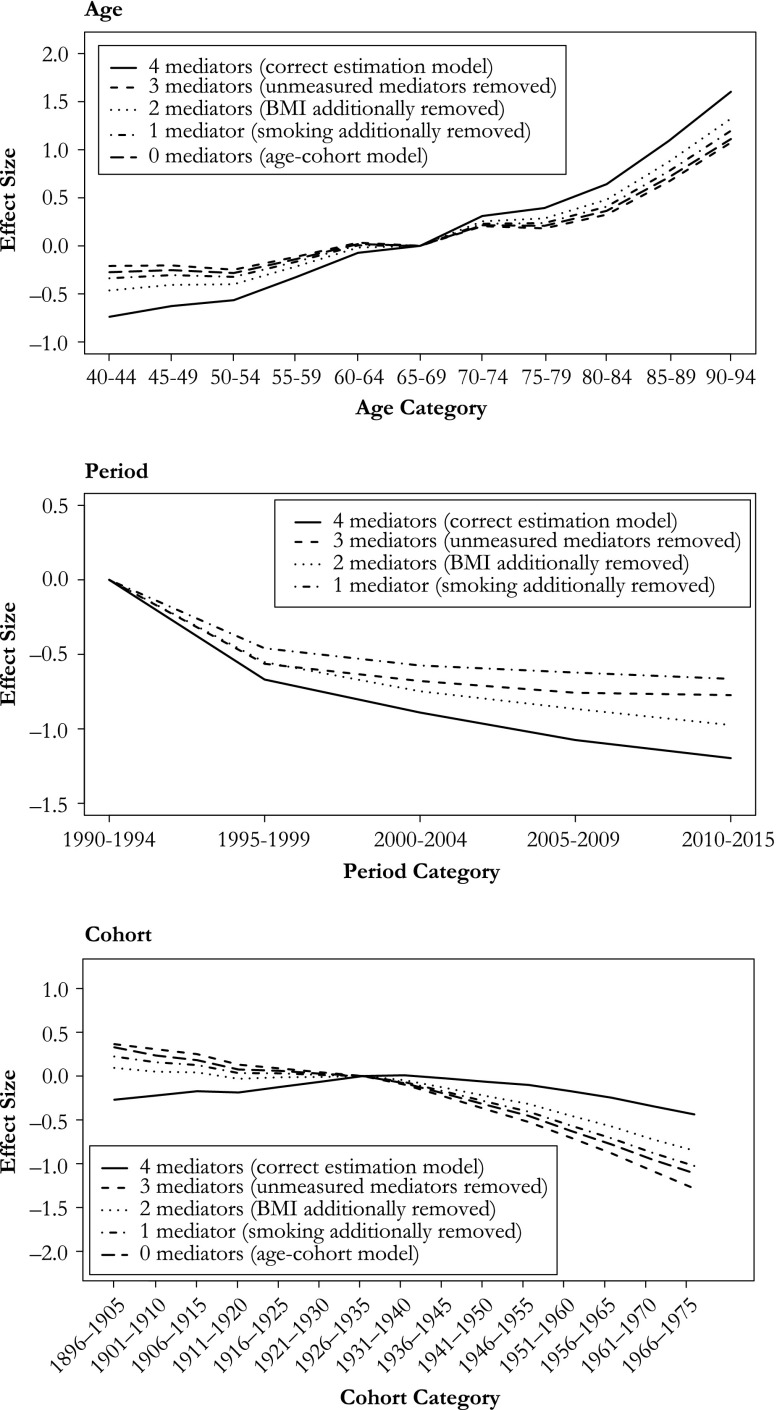
Average estimated parameters for the APC effects in Scenario 1 (*simple*) using the mechanism-based approach. Summary of 1,000 simulations

Removing mediators from the estimation of the period path introduces bias (Fig. 4 for probit variant, see Fig. S3 in Online Resource 2 for logistic variant). The relative magnitude of the bias appears strongest for birth cohort, which had weak negative parameter estimates when the model was correctly specified, but these estimates become strongly negative when mediators are removed from the estimation model. The directions of the bias are as expected (see the earlier section, Linear Dependency and Expected Bias). On average, removing the *unmeasured* period mediators has a negative effect on the estimated age and cohort effects, while it introduces a positive bias on the period estimates; the estimated parameters for the age and cohort effects become less positive, while those for the period effect become less negative. The same occurs when smoking and statins are additionally removed. This is as expected because the paths via the *unmeasured* period mediators, smoking, and statins all have a negative effect on the outcome (Table 1). Removing BMI leads to the opposite: that is, it introduces a positive bias on the estimated effects of age and cohort, and a negative one on that of period. Again, this is as expected because the path from *P* to *Y* through BMI is positive (Table 1).

### Scenario 2: *More Causes*

**Fig. 5:**
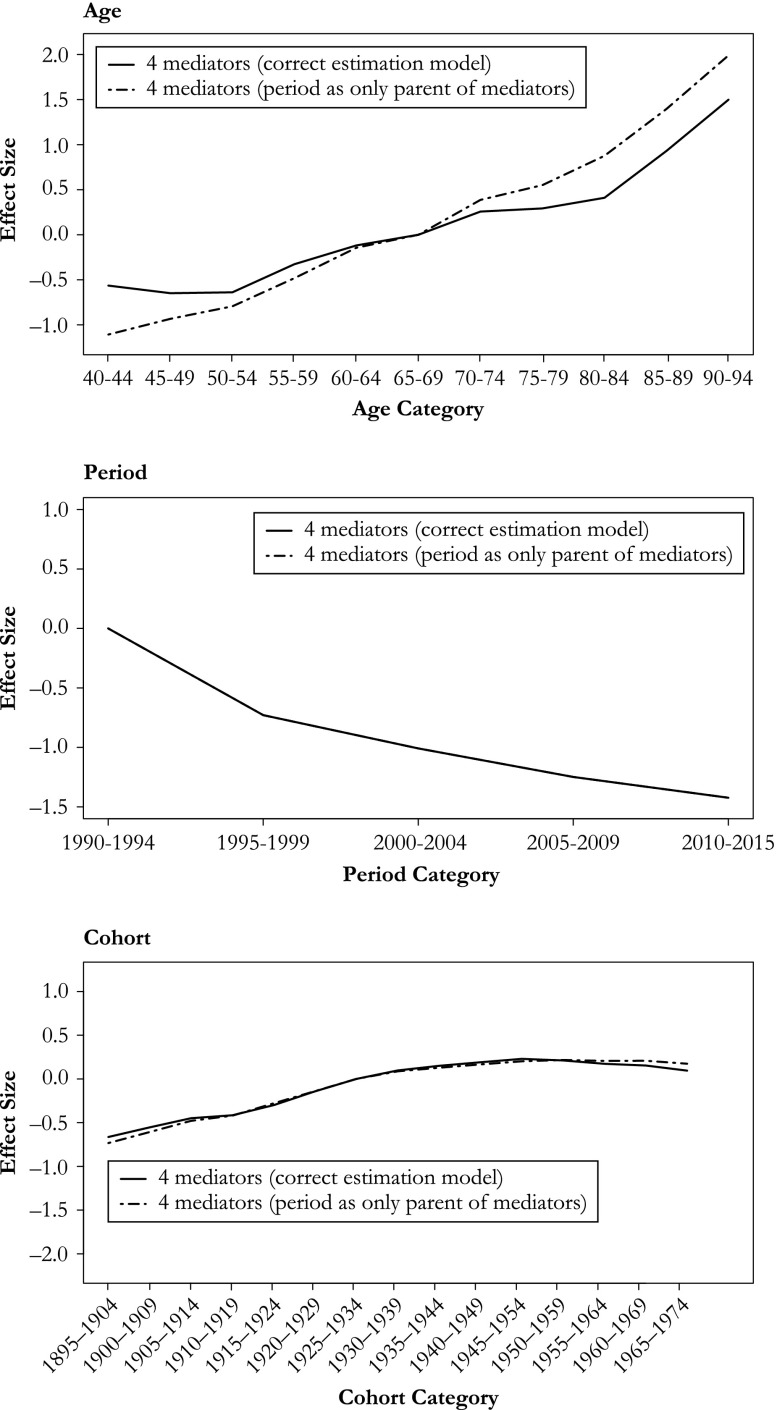
Average APC estimates in Scenario 2 (*more causes*) using the mechanism-based approach. Summary of 1,000 simulations

In the scenario in which age (in addition to period) is a cause of BMI, and cohort (in addition to period) is a cause of smoking, we find that not including these relationships in the estimation model also results in bias. As expected, this bias is largest for age, where the age effect is overestimated when the negative effect of age on BMI is not included in the estimation model (Fig. 5). A similar but much weaker bias is found for cohort when the effect of *C* on smoking is not included (Fig. 5 for the probit variant; Fig. S4 in Online Resource 2 for logistic variant). There is no bias in the estimation of the period effect (Fig. 5), except in the logistic variant (Fig. S4).

These results follow our expectation. The effect of age on the outcome via BMI is negative. Because age is not included as a cause of BMI in the estimation model, this negative effect is subtracted from the total age effect, thereby resulting in an overestimation of the age effect. The effect of not modeling birth cohort as a cause of smoking follows the same logic, but the bias is weaker because the effect size from birth cohort to the outcome via smoking is also smaller. In this scenario, the period effect estimates are not biased because period is correctly modeled as a cause of the four mediators. The logistic variant is nevertheless sensitive to this bias because of the nonlinearities in its estimation procedure. Finally, removing mediators from the estimation model results in biases in the same direction as found in the *simple* scenario (see Online Resource 2, Figs. S1 and S4), and the same explanations for these directions apply.

### Scenario 3: *Confounding*

**Fig. 6:**
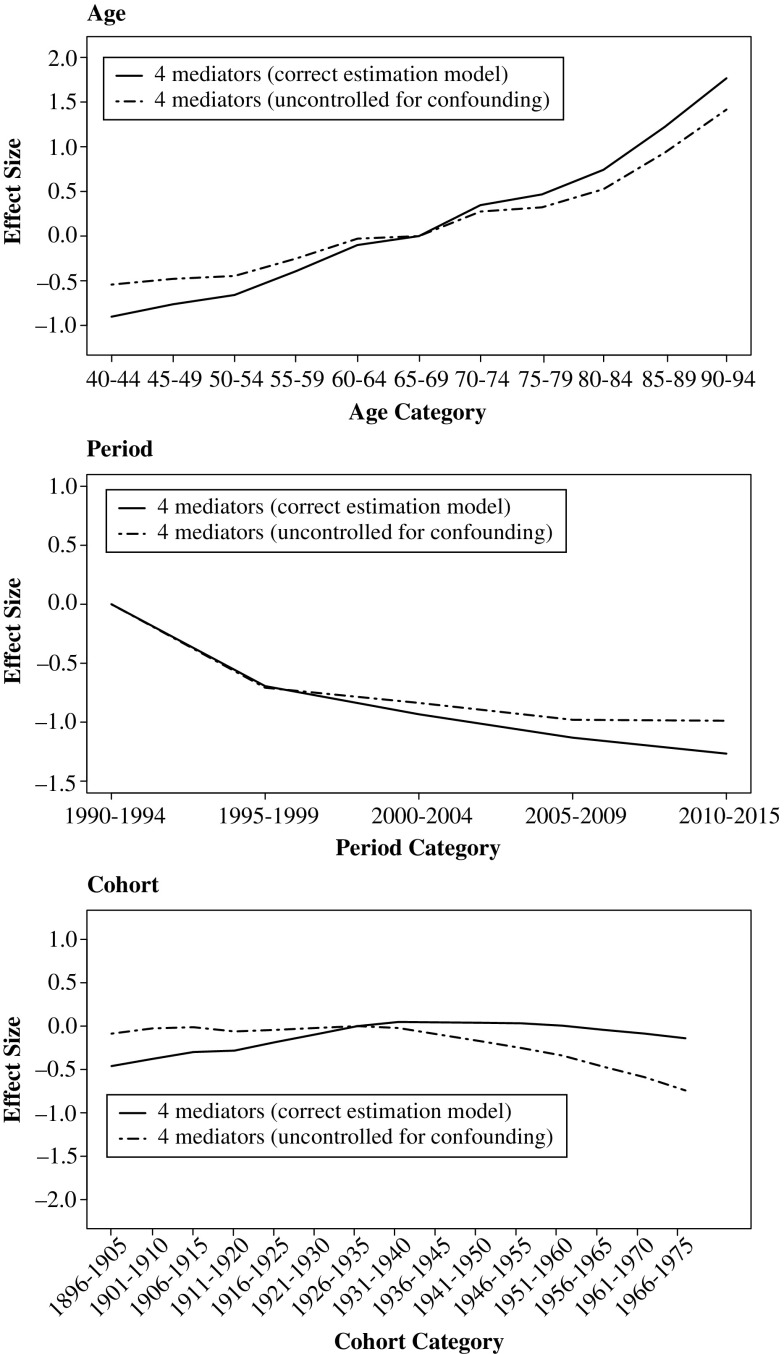
Average APC estimates in Scenario 3 (*confounding*) using the mechanism-based approach. Summary of 1,000 simulations

In the scenario of a variable (genotype) confounding the relationships between BMI and CVD death, and between smoking and CVD death (both mediators on the *P*–*Y* path), we find that failing to control for this confounder results in bias in all three age, period, and cohort effect estimates. The age parameter estimates become somewhat negatively biased when we do not control for genotype in our outcome model (Fig. 6 for the probit variant; Online Resource 2, Fig. S5 for the logistic variant). The same occurs in the cohort effect, while the period effect suffers from a small positive bias (Fig. 6).

The bias observed here is caused by collider stratification (Cole et al. 2010; Elwert and Winship 2014). Collider stratification bias—also known as *endogenous selection*—occurs when two variables both have causal effects on a third variable (the *collider*), and the collider is conditioned upon (Elwert and Winship 2014; Greenland 2003). Doing so creates an artificial association between the two causes of the collider that is of sign opposite to the product of the signs of the effects into the collider. In traditional APC analysis, collider stratification does not occur because APC effects on an outcome are estimated without adjusting for confounders or mediators.

In the scenario considered here, genotype has a positive causal effect on BMI, on smoking, and on CVD mortality. Therefore, BMI and smoking are colliders in the paths between *P* and genotype. By including BMI and smoking (together with the other two mediators) in our outcome model, we create an additional spurious association between *A* and *Y* via *A* – *P* – BMI – genotype – *Y*, and one between *C* and *Y* via the path *C* – *P* – smoking – genotype – *Y*. Both of these spurious associations are negative because those induced by collider stratification—namely, *P*-BMI-genotype and *P*-smoking-genotype—are both negative, while *A* – *P*, *C* – *P*, and genotype – CVD are all positive.

### Varying Effect Sizes

**Fig. 7:**
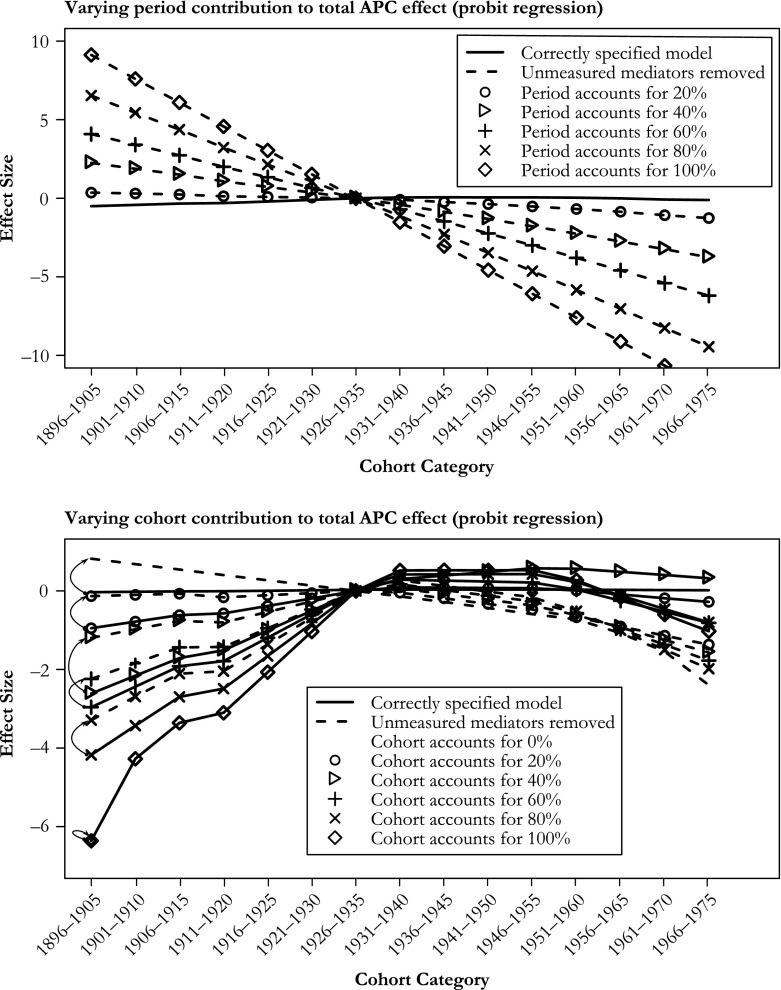
Varying the effect sizes of period from 0 % to 100 % of the total APC effect in 20 % increments while keeping cohort effect constant (upper), varying the effect sizes of cohort from 0 % to 100 % of the total APC effect in 20 % increments while keeping period effect constant (lower). When period accounts for 100 %, the correctly specified cohort trend is a horizontal line at *y* = 0. No bias when cohort accounts for 100 % because then the period effect (source of bias) accounts for 0 %. Only cohort figures (probit variant) are shown. Arrows in lower figure indicate the size and direction of the bias

Increasing the size of the period effect and keeping the size of the cohort effect constant resulted in increased bias when the *unmeasured* mediator was removed from the estimation model (Fig. 7 for the probit variant, Figs. S6 and S7 in Online Resource 2 for the logistic and continuous variants). Keeping the period effect constant and increasing the size of the cohort effect resulted in roughly equal amounts of bias in the cohort effect estimate (Fig. 7 and Figs. S6 and S7 in Online Resource 2); the bias was equal in the continuous variant because that estimation is done without link function transformations.

## Discussion

We assessed the performance of mechanism-based APC models in realistic settings and extended the method to incorporate all generalized linear models, as well as nonlinearities in the predictors, using Monte Carlo simulation. We found that in simple scenarios in which a single set of mediators for *P* is not affected by unmeasured confounding, the mechanism-based approach (extended or not) performed reasonably well for the estimation of all three age, period, and cohort effects, especially if the mediators that were not included in the estimation had opposite signs (e.g., *unmeasured* and BMI) so that their unmeasured contributions (partially) cancelled out. The bias we observed was in line with our expectations derived from the APC linear dependency. In the scenarios with additional complications—that is, either when mediators were caused by more than one of three APC components, or there was unmeasured confounding of the mediator-outcome relationships—we found additional bias. This, again, was in line with our expectations. Findings were similar for the probit and logistic variants, although we did not directly compare these variants because of their differences in transforming effects into probabilities.

### (Un)testable Assumptions

APC models solve the linear dependency problem by imposing modeling constraints by fiat (Fienberg 2013). The mechanism-based approach does not differ from this because it is (commonly, as explained in the next paragraph) not possible to identify from the data whether a variable is a mediator for age, period, cohort, a combination of two of these, or all three variables. This is, of course, the same problem that occurs in any conventional APC analysis when attempting to decompose some outcome into age, period, and cohort effects. Therefore, the untestable assumptions that are made in conventional APC analysis move to the mediator stage of the modeling procedure.

The addition of mediators to the APC model can be tested (Winship and Harding 2008). We omitted this test from our assessment because the test is conditional on having (at least) a full set of mediators for one of the three APC variables. We consider it likely that in the majority of real-life applications, it will not be possible to find a full set of mediators for even one of the APC variables, and hence we focused our assessment on possible biases that may be encountered, such as those due to missing mediators.

### Simulating Bias

In our simulations, we considered four mediators on the period path and kept the *more causes* scenario separate from the *confounding* scenario in order to illustrate, separately, possible pitfalls that may be encountered when the mechanism-based approach is used. In a real application of the method, many more mediators may exist, which may also have more than one cause, and their relation with the outcome may be confounded. However, a more complicated scenario need not necessarily result in more biased estimates because biases of opposed sign may cancel each other out, such as when BMI was removed from the estimation model in our simulations (because BMI was the only positive mediator). Most importantly, when potential bias is assessed, it is the size of the omitted pathways that matters, relative to the size of the included pathways. This was demonstrated in our analysis varying the size of the period effect and omitting a period mediator from the estimation model: a larger period effect resulted in a larger bias, whereas keeping the size of the period effect constant and increasing the size of the cohort effect resulted in roughly the same magnitude of bias and smaller relative bias. Analogous simulations where instead age or cohort mediators had been removed would have yielded the same conclusions; only the sign of the bias would differ as shown in the earlier section on linear dependency and expected bias.

### Confounding and Colliding

Because the mechanism-based approach uses a mediation approach to APC analysis, it is also subject to biases that are not present in traditional APC analysis: (1) confounding of the mediator-outcome relationship, and (2) collider stratification bias (Elwert and Winship 2014; Greenland 2003).

Arguably, traditional APC analysis is not affected by confounding because age, period, and cohort are time dimensions and therefore are not causally affected by other variables. In our *confounding* scenario, the effect of BMI (and smoking) on CVD mortality was confounded by genotype, which thereby also affected the estimation of the period effect. Ideally, such a confounder would be controlled. However, if the confounder is unmeasured—and therefore cannot be controlled—a difficult choice has to be made. Removing the mediator induces bias via an omitted pathway, whereas including the mediator induces confounding bias. Confounding of the mediator-outcome relationship (and hence collider stratification bias) resulted in more bias than omitting the relevant pathway in our simulations, but this is dependent on the relative strengths of mediation and confounding.

In our *confounding* simulations, BMI was affected by period and by genotype (as was smoking). Because of the linear dependency of period with age and cohort, collider stratification bias also affected the age and cohort estimates. Also in this scenario, conditioning on genotype would have prevented this bias. If conditioning on both causes of the collider is not possible, a choice has to be made between removing and keeping the mediator in the analysis. Greenland’s (2003) second rule of thumb regarding endogenous selection states that including mediators that are also colliders induces larger bias than the size of the mediator’s dependence on its causes (Elwert and Winship 2014). This suggests removing such mediators from the analysis, despite inducing bias via omitted pathways.

### Directed Acyclic Graphs (DAG)

We encourage investigators to draw causal DAGs of the relations among APC variables, mediators, and outcome. To represent the deterministic relationship among age, period, and cohort in the causal DAG, we used bold arrows, which is in line with conventions in causal inference (Spiegelhalter et al. 2002; Spirtes et al. 2001) but differs from the representation used by Winship and Harding (2008). We used these arrows because the relationships among age, period, and cohort are fundamentally different from those of other relationships in the DAG (which are stochastic and causal rather than deterministic).

By drawing the relations between APC and mediators, we are clear about the assumptions underlying our analyses. Such clarity sets the mechanism-based approach apart from other APC approaches, where transparency about constraints can be lacking (Luo 2013). Drawing causal DAGs and redrawing them after one of the three age, period, or cohort variables is removed (Fig. 3) helps explicate otherwise hidden assumptions about the relationships among age, period, cohort, and their mediators and can help identify possible biases, such as collider stratification bias.

### Guidelines for Application of the Mechanism-Based Approach

Based on our assessment, when estimating age, period, and cohort effects by applying the mechanism-based approach (extended or not), we suggest considering the following questions:

1. Which APC variable is believed to have the weakest effect on the outcome in question?
2. Which APC variable has putative mediators available in the data?
3. How well measured are these mediators?
4. How exhaustive are these mediators of all the pathways linking the respective APC variable and the outcome?
5. Are there common causes of these mediators (e.g., is more than one APC variable affecting the mediators)?
6. Are mediator-outcome relationships potentially confounded, and how severely?

To minimize bias, select the APC variable for which the answers to questions 1–6 are most favorable. For this variable, model the mediated pathways; the other two effects can be modeled directly. The degree of bias that remains in the analysis is dependent on the answers to the preceding questions for the chosen APC variable and on ordinary modeling concerns, such as correct modeling of the functional form of the relationships among APC variables, mediators, and outcome.

Answering questions 1–6 will likely require making assertions that cannot be tested in the data, and is thereby similar to setting constraints by fiat like in various other APC approaches. However, the difference is that the answers to these questions—particularly, questions 1, 4, 5, and 6—can be motivated based on substantive theoretical reasoning. As described in our Introduction, various authors have argued that age-period-cohort analysis needs to become more theoretically informed. A large suite of APC methods exist that solve the linear identification problem nontransparently and without substantive theoretical justification. A strength of the mechanism-based approach is that it motivates researchers to be explicit about their (substantive theoretical) assumptions.

## Conclusion

We demonstrated the performance of the mechanism-based approach to APC modeling in nonideal circumstances. Biases occurred when the assumed causal relations did not coincide with the truth, such as when paths from causes to mediators were omitted from the estimation model, or when there was unmeasured confounding of the mediator-outcome relationship. The direction of the bias followed our expectations based on APC linear dependency. Size of bias is dependent on the size of the effects involving confounders, omitted intermediate variables, or pathways. Our extension of the mechanism-based approach increases its utility by allowing it to be easily usable for models with nonlinear link functions and parameterizations of any complexity. Our brief guidelines, aided by causal DAGs, offer a useful tool for researchers who wish to implement this approach.

## Electronic supplementary material

ESM 1 (R 7 kb)

ESM 2 (DOCX 1186 kb)
