# Supplementary material for: An Assessment and Extension of the Mechanism-Based Approach to the Identification of Age-Period-Cohort Models
Source: Demography. 2017 Mar 9;54(2):721–43. doi: 10.1007/s13524-017-0562-6 (PMC5371644; doi:10.1007/s13524-017-0562-6)
Supplement: Supplementary file 2 — (DOCX 1186 kb) [file 13524_2017_562_MOESM2_ESM.docx]

**Online Resource 2**

**An Assessment and Extension of the Mechanism-Based Approach to the Identification of Age-Period-Cohort Models**

Maarten J. Bijlsma, Rhian M. Daniel, Fanny Janssen, and Bianca L. De Stavola

**Example of the Path Tracing Rule**

Consider the situation where we seek to estimate the effects of A, P and C on body weight in women (Y). Assume the two mediators, daily caloric intake (M_1_) and daily calories burned (M_2_), completely mediate the effect of P on Y, and (somewhat questionably perhaps) that these mediators are not affected by either A or C.

In the first step we estimate the effect of period separately on the two mediators, here using linear regression,

E(M_1_| P) = $\gamma_{0}+\gamma_{1}\cdot P$

E(M_2_| P) = $\gamma_{2}+\gamma_{3}\cdot P$

The period effect on $M_{1}$ is then represented by $\gamma_{1}$, and the period effect on $M_{2}$ by $\gamma_{3}$.

Correct specification of the above equation is sufficient for unbiased estimation of these parameters by ordinary least squares (OLS). For unbiased estimation of the precision of these parameters using the conventional variance estimator routinely used alongside OLS, we assume that the errors ε_i_ = M _i_ - E(M_1_ | P_i_), i=1, …, n, are independent and homoskedastic. These additional conditions could be relaxed if instead we used a sandwich estimator of variance.

In the second step we estimate the effect of age, the two period mediators, and cohort on body weight using a linear regression model,

E($Y|A,M_{1},M_{2},C)= \delta_{0}+ \delta_{1}\cdot A + \delta_{2}\cdot C+{\delta_{3}\cdot M_{1}+\delta_{4}\cdot M}_{2}$

The age effect on Y is then represented by $\delta_{1}$, and the cohort effect by $\delta_{2}$.

Again, unbiased estimation of these parameters by OLS follows from the correct specification of the forms of the conditional expectations given above. Again, the conventional variance estimators rely on independent homoskedastic errors for unbiased estimation, violations of which can be accommodated using sandwich estimators.

Using the path tracing rule, the effect of P on Y through M_1_ is equal to $\gamma_{1}\cdot\delta_{3}$ and the effect of P on Y through M_2_ is equal to $\gamma_{3}\cdot\delta_{4}$ Hence the total effect of P on body weight is equal to $\gamma_{1}\cdot\delta_{3}$ + $\gamma_{3}\cdot\delta_{4} .$

**Estimating APC effects using the mechanism-based approach when more than one of APC affects a mediator, or if there are multiple mediators**

In scenario 2 ‘more causes’, next to period, age was an additional cause of BMI, and cohort was an additional cause of smoking. Hence, these mediators had more than one cause. Furthermore, in all scenario’s, we had four mediators on the path from APC to the outcome. Therefore, we will explain estimation in a setting where there are more causes of mediators, and where there is more than one mediator, using scenario 2. We will describe estimation both in the probit variant (using the multiplication approach) and the logistic variant (using the Monte Carlo approach). We will again use a linear representation for ease of reading, but in our simulations we modelled age, period and cohort as categorical variables through dummy coding.

*Multiplication approach*

First, we estimated a probit model with age A, cohort C, Unmeasured (M_1_)_,_ BMI (M_2_)_,_ Smoking (M_3_)_,_ and Statins (M_4_)_:_

*E(Y | A, M_1_, M_2_, M_3,_ M_4,_ C) =* $\Phi(\delta_{0}+\delta_{1}$ *· A +* $\delta_{2}$ *· C +* $\delta_{3}$*· M_1_ +* $\delta_{4}$*· M_2_ +* $\delta_{5}$*· M_3_ +* $\delta_{6}$*· M_4_)*

Where $\Phi$ is the probit link. In this case, $\delta_{1}$and $\delta_{2}$only represent partial effect estimates of the age and cohort effects, respectively, because part of the age and cohort effects go through the mediators. The *δ*’s 3 through 6 represent the effect of the mediators on the outcome.

Secondly, we modelled the mediators, using their causes as covariates in our models:

*E(*$M_{1}$*| P) =* $\gamma_{0}$ + $\gamma_{1}\cdot$ P + $\nu_{1}$

*E(*$M_{2}$*| A, P) =* $\gamma_{2}$ + $\gamma_{3} \cdot A+\gamma_{4} \cdot$ P + $\nu_{2}$

*E(*$M_{3}$*| C, P) =* $\Phi(\gamma_{5}$ *+* ${\gamma_{6} \cdot C+\gamma}_{7}\cdot$*P)*

*E(*$M_{4}$*| P) =* ${\Phi(\gamma}_{8}$ *+* $\gamma_{9}\cdot$*P)*

The total age effect was estimated as: $\delta_{1}+\gamma_{3}\cdot\delta_{4}$

The total period effect was estimated as: $\delta_{3}\cdot\gamma_{1}+ \delta_{4}\cdot\gamma_{4}+ \delta_{5}\cdot\gamma_{7}+ \delta_{6}\cdot\gamma_{9}$

The total cohort effect was estimated as: $\delta_{2}+\gamma_{6}\cdot\delta_{5}$

We applied the bootstrap, and used 2.5% and 97.5% quantiles from the bootstrap distribution to find confidence intervals for the estimates of age, period and cohort parameters.

*Monte Carlo approach*

In a first step, we estimated the mediators Unmeasured (M_1_)_,_ BMI (M_2_)_,_ Smoking (M_3_)_,_ and Statins (M_4_)_,_ using the following models:

$M_{1}\left( P \right)=\gamma_{0}+\gamma_{1}\cdot P+$ $\varepsilon_{3}$

$M_{2}(A,P)=\gamma_{2}+{\gamma_{3}\cdot A+ \gamma}_{4}\cdot P$ $\boldsymbol{+}\varepsilon_{4}$

$$logit\{E[M_{3}\left( C,P \right)]\}=\gamma_{5}+\gamma_{6}{\cdot C+ \gamma}_{7}\cdot P$$

$$logit\{{E[M}_{4}\left( P \right)]\}=\gamma_{8}+\gamma_{9}\cdot P$$

In the second step, we estimated the outcome:

$$logit\{E[Y\left( A,M_{1},M_{2},M_{3},M_{4},C \right)]\}= \delta_{0}+ \delta_{1}\cdot A +\delta_{2}\cdot M_{1} +\delta_{3}\cdot M_{2} +\delta_{4}\cdot M_{3} +\delta_{5}\cdot M_{4} + \delta_{6}\cdot C$$

In the third step, we simulated mediators $\tilde{M_{1}}$*(*$\tilde{p})$, $\tilde{M_{2}}$*(*$\tilde{p}), \tilde{M_{3}}$*(*$\tilde{p})$ and $\tilde{M_{4}}$*(*$\tilde{p})$. First we independently generated a range of age values $\tilde{a}$, period values $\tilde{p}$, and cohort values $\tilde{c}$, which had the same range as empirically observed age, period and cohort values (but $\tilde{a}$ = $\tilde{p}$ – $\tilde{c}$ does not hold for the generated values). For the continuous variables, we could then generate mediator values immediately, using the estimates from the first step:

$$\tilde{M_{1}}(\tilde{p}) =\hat{\gamma}_{0}+\hat{\gamma}_{1}\cdot\tilde{p}+e$$

$$\tilde{M_{2}}\left( \tilde{a},\tilde{p} \right) =\hat{\gamma}_{2}+\hat{\gamma}_{3}\cdot\tilde{a}+\hat{\gamma}_{4}\cdot\tilde{p}+e$$

For the binary variables, we first estimated means

$$\hat{\mu}_{\tilde{m}3,\tilde{p,}\tilde{c}}= \frac{exp(\hat{\gamma}_{5}+\hat{\gamma}_{6}\cdot\tilde{c}+\hat{\gamma}_{7}\cdot\tilde{p})}{1+exp(\hat{\gamma}_{5}+{\hat{\gamma}_{6}\cdot\tilde{c}+\hat{\gamma}}_{7}\cdot\tilde{p})}$$

$$\hat{\mu}_{\tilde{m}4,\tilde{p}}= \frac{exp(\hat{\gamma}_{8}+\hat{\gamma}_{9}\cdot\tilde{p})}{1+exp(\hat{\gamma}_{8}+\hat{\gamma}_{9}\cdot\tilde{p})}$$

And then drew from Bernoulli distributions with these means.

In the fourth step, we simulated values $\tilde{Y}(\tilde{a}, \tilde{p},\tilde{c})$for the outcome using the estimates from step 2 and the simulated mediator values from step 3. We drew from a Bernoulli distribution with mean

$$\hat{\mu}_{y, \tilde{a},\tilde{p},\tilde{c}}= \frac{\exp{(\hat{\delta}}_{0}+\hat{\delta}_{1}\cdot\tilde{a}+\hat{\delta}_{2}\cdot\tilde{M_{1}}+\hat{\delta}_{3}\cdot\tilde{M_{2}}+\hat{\delta}_{4}\cdot\tilde{M_{3}}+\hat{\delta}_{5}\cdot\tilde{M_{4}}+\hat{\delta}_{6}\cdot\tilde{c})}{1+exp(\hat{\delta}_{0}+\hat{\delta}_{1}\cdot\tilde{a}+\hat{\delta}_{2}\cdot\tilde{M_{1}}+\hat{\delta}_{3}\cdot\tilde{M_{2}}+\hat{\delta}_{4}\cdot\tilde{M_{3}}+\hat{\delta}_{5}\cdot\tilde{M_{4}}\hat{\delta}_{6}\cdot\tilde{c})}$$

In the fifth step, we fit a logistic regression model to $\tilde{Y}(\tilde{a}, \tilde{p},\tilde{c})$ with $\tilde{a}$*,* $\tilde{p}$ and $\tilde{c}$ as covariates.

We applied the bootstrap, and used 2.5% and 97.5% quantiles from the bootstrap distribution to find confidence intervals for the estimates of age, period and cohort parameters.

**Additional Figures**

**
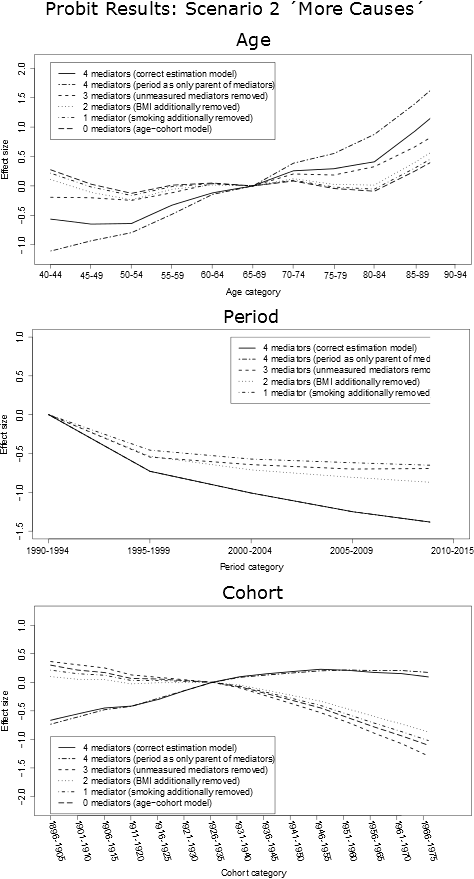
**

**Figure S1.** Average estimated parameters for the age, period and cohort effects in scenario 2 (More causes) probit variant using the mechanism-based approach: summary of 1000 simulations.


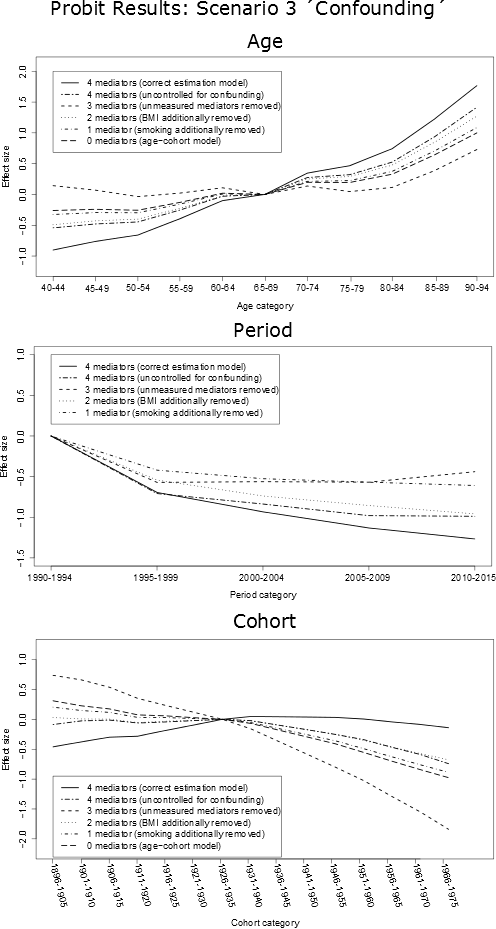


**Figure S2.** Average estimated parameters for the age, period and cohort effects in scenario 3 (Confounding) probit variant using the mechanism-based approach: summary of 1000 simulations.

**
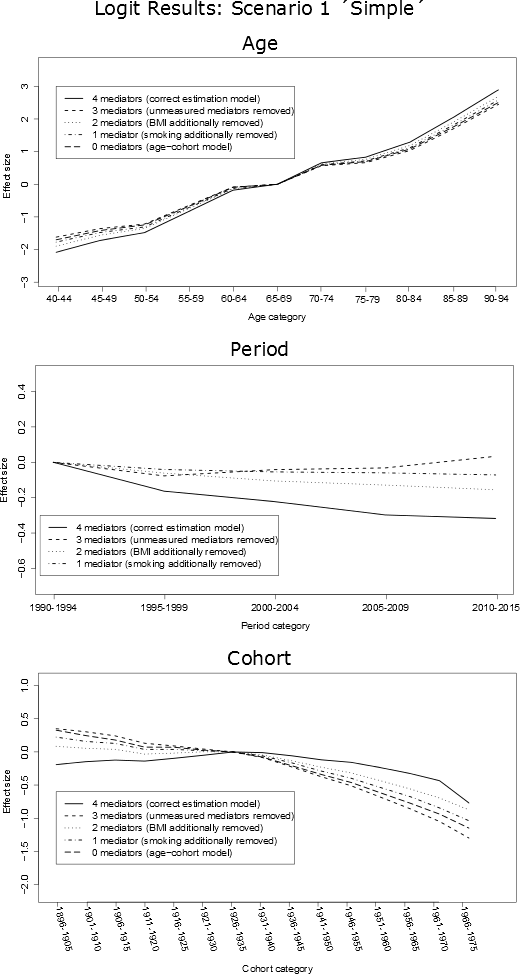
**

**Figure S3.** Average estimated parameters for the age, period and cohort effects in scenario 1 (Simple) logistic variant using the mechanism-based approach: summary of 1000 simulations.

**
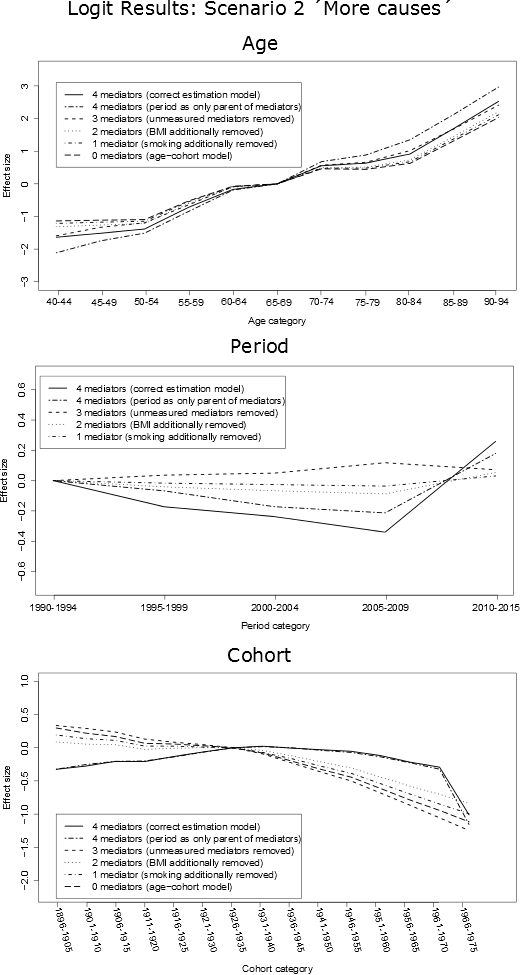
**

**Figure S4.** Average estimated parameters for the age, period and cohort effects in scenario 2 (More causes) logistic variant using the mechanism-based approach: summary of 1000 simulations.


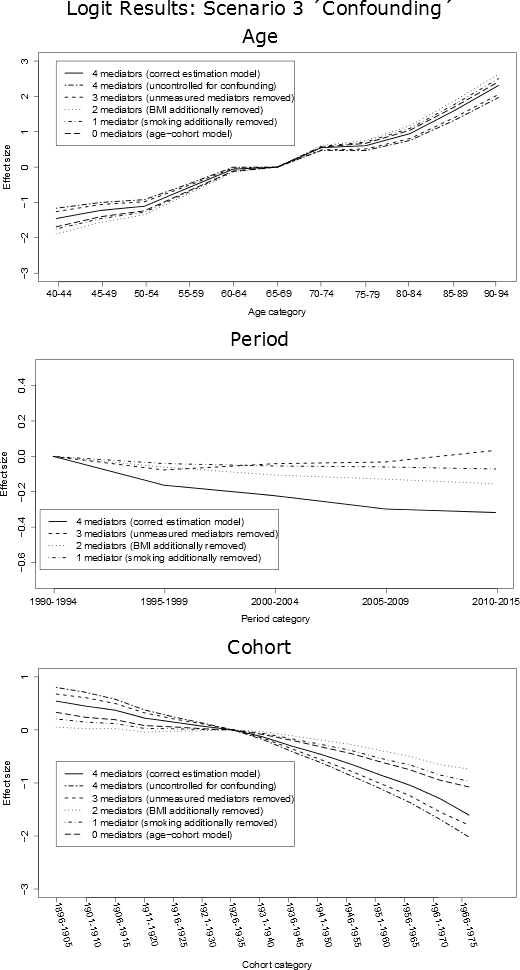


**Figure S5.** Average estimated parameters for the age, period and cohort effects in scenario 3 (Confounding) logistic variant using the mechanism-based approach: summary of 1000 simulations.

**Varying age, period and cohort contributions**

To demonstrate how the unequally distributed strength of age, period and cohort affect the bias, we performed two sets of simulations in which we vary these strengths. In the first set, we vary the size of the period effect from 0 to 100% in 20% increments, while correspondingly reducing the size of the age effect, and keeping the size of the cohort dimension constant at 10% (excluding the last increment, where cohort is necessarily set to 0%). The second set is identical, but then the cohort effect size is varied and period kept constant at 20%. In both sets, bias is generated by removing the first mediator (‘unmeasured’) from the estimation model in the ‘simple’ scenario. These simulations were done with probit, logit and linear regression variants. The linear regression variant is identical to the probit variant, with the exception that the probit transformation was not used when generating the data and estimating from the data. The probit variant of the first two sets can be found in the main document. A third set of simulations in which the age effect size is varied is described later in this supplemental document.


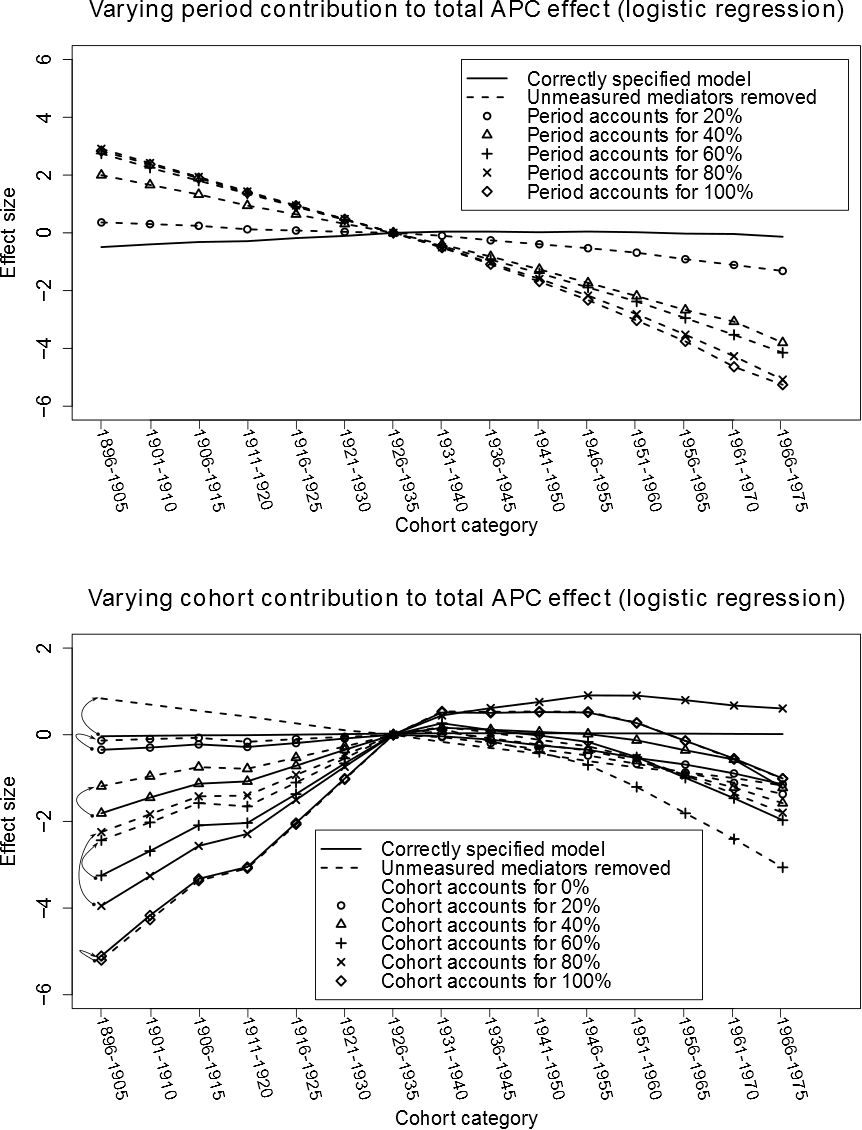


**Figure S6.** Varying the effect sizes of period from 0 to 100% of the total APC effect in 20% increments while keeping cohort effect constant (upper), varying the effect sizes of cohort from 0 to 100% of the total APC effect in 20% increments while keeping period effect constant (lower). When period accounts for 100% the correctly specified cohort trend is a horizontal line at y=0. No bias when cohort accounts for 100% because then the period effect (source of bias) accounts for 0%. Only cohort figures (logistic variant) shown. Arrow in lower figure indicates the size and direction of the bias.

**
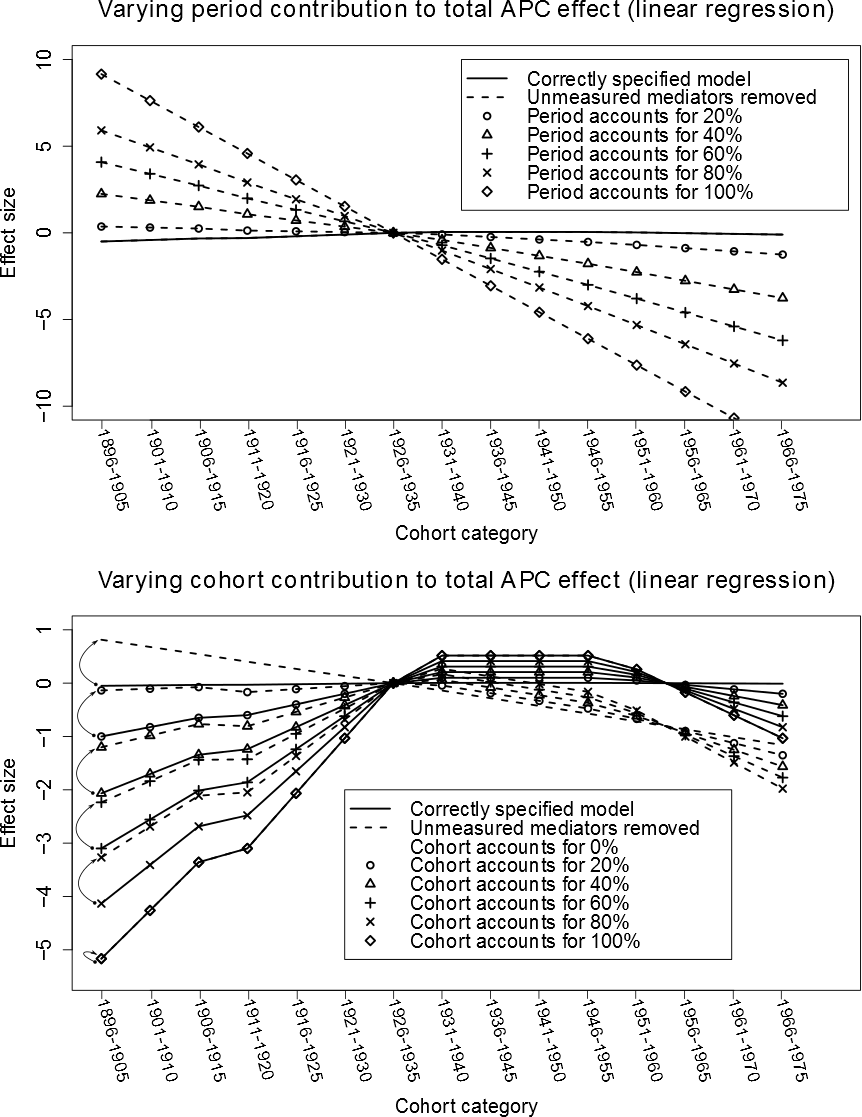
**

**Figure S7.** Varying the effect sizes of period from 0 to 100% of the total APC effect in 20% increments while keeping cohort effect constant (upper), varying the effect sizes of cohort from 0 to 100% of the total APC effect in 20% increments while keeping period effect constant (lower). When period accounts for 100% the correctly specified cohort trend is a horizontal line at y=0. No bias when cohort accounts for 100% because then the period effect (source of bias) accounts for 0%. Only cohort figures (linear regression variant) shown. Arrow in lower figure indicates the size and direction of the bias.

**Varying the age contribution to the total APC effect**

We vary the size of the age effect from 0 to 100% in 20% increments, while correspondingly reducing the size of the cohort effect (changing period, the source of bias, would result in an additional source of variance and so we choose cohort), and keep the size of the period dimension constant at 20% (excluding the two last increments, where period has to be set to 0). Bias is generated by removing the first mediator (‘unmeasured’) from the estimation model in the ‘simple’ scenario. These simulations were done with probit, logit and linear regression variants. The linear regression variant is identical to the probit variant, with the exception that the probit transformation was not used when generating the data and estimating from the data.

See next page for the figure.

**
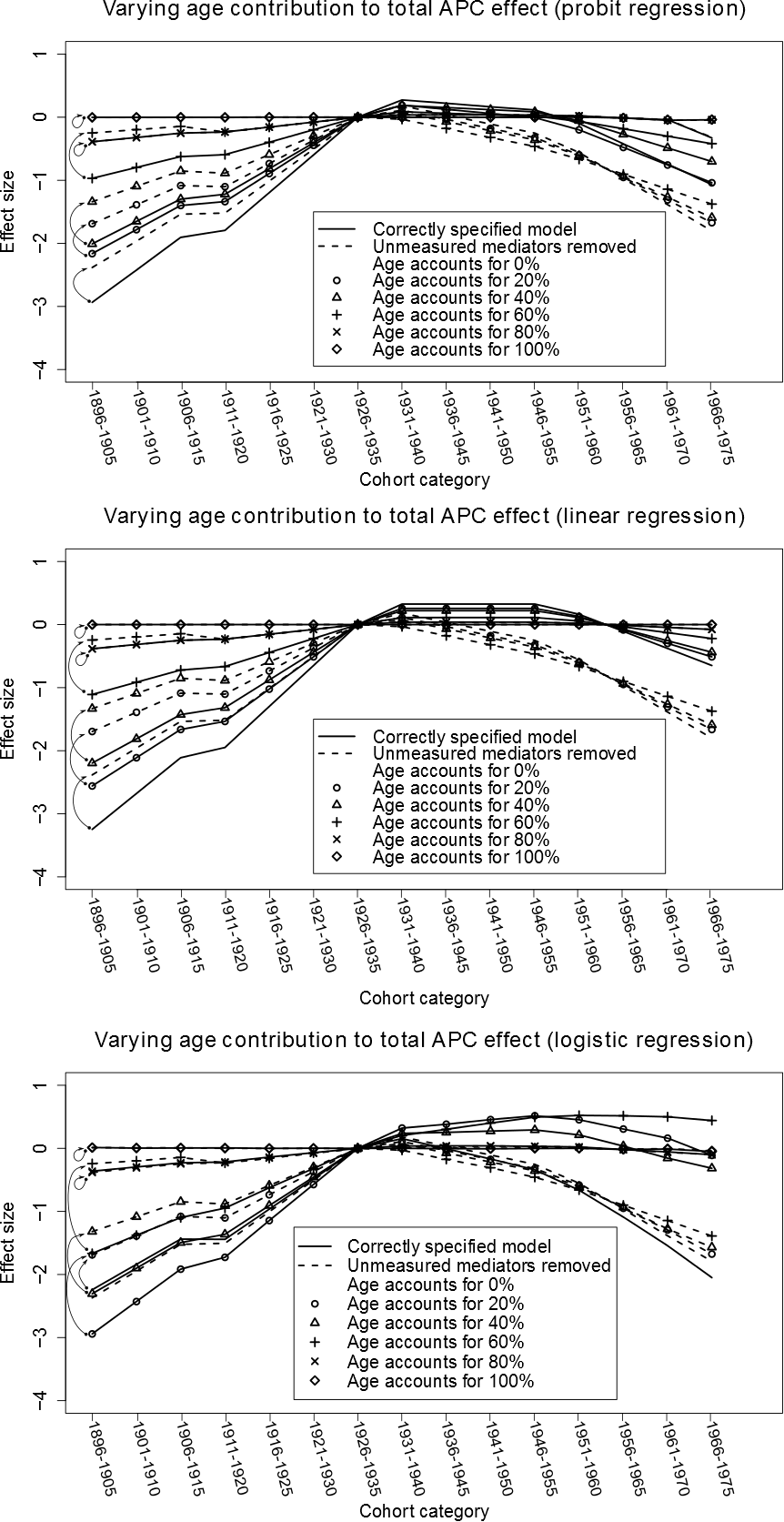
**

**Figure S8.** Varying the effect size of age from 0 to 100% of the total APC effect in 20% increments following description on previous page. Logistic transformation differs from probit and linear regression estimation because of the logistic (nonlinear) transformation.

**Traditional age-period-cohort analysis**

There are many approaches to age-period-cohort analysis, some formulated decades ago but nevertheless still relevant (e.g. Fienberg and Mason, 1976; Hobcraft et al. 1982; Holford, 1983; Clayton & Schifflers, 1986). Arguably, an advantage of these methods is that they place relatively simple constraints on the APC model, and therefore the direction of bias may also be easier to identify. For completeness, we here report the results of such an APC analysis. In particular, this model is applied to exactly the same data as the data produced in scenario 1 ‘simple’ (probit variant).

*Model specification*

The model is specified as a probit regression model with terms for age, period and birth cohort. The model becomes identifiable by constraining two age, period or cohort categories to be equal (Clayton & Schifflers, 1986). This can be easily be achieved by setting two age, period or cohort categories as reference categories (instead of one category for each); i.e. when a dummy specification is used for the categories, this can be done by removing an additional dummy. In particular, we fitted two such models; one where the parameters for the 1931-1939 and the 1936-1944 birth cohorts were set to be equal, and one where those for the 1990-1995 and the 1995-2000 periods were set to be equal.

*Results*

Fitting the model with constraints on the birth cohort parameters leads to good average estimates of the age effect, very poor average estimates of the period effect and quite good estimates of the average cohort effect (Figure S8). On the other hand, the model with constraints on the period parameters has strongly biased estimates of all three effects (Figure S8).


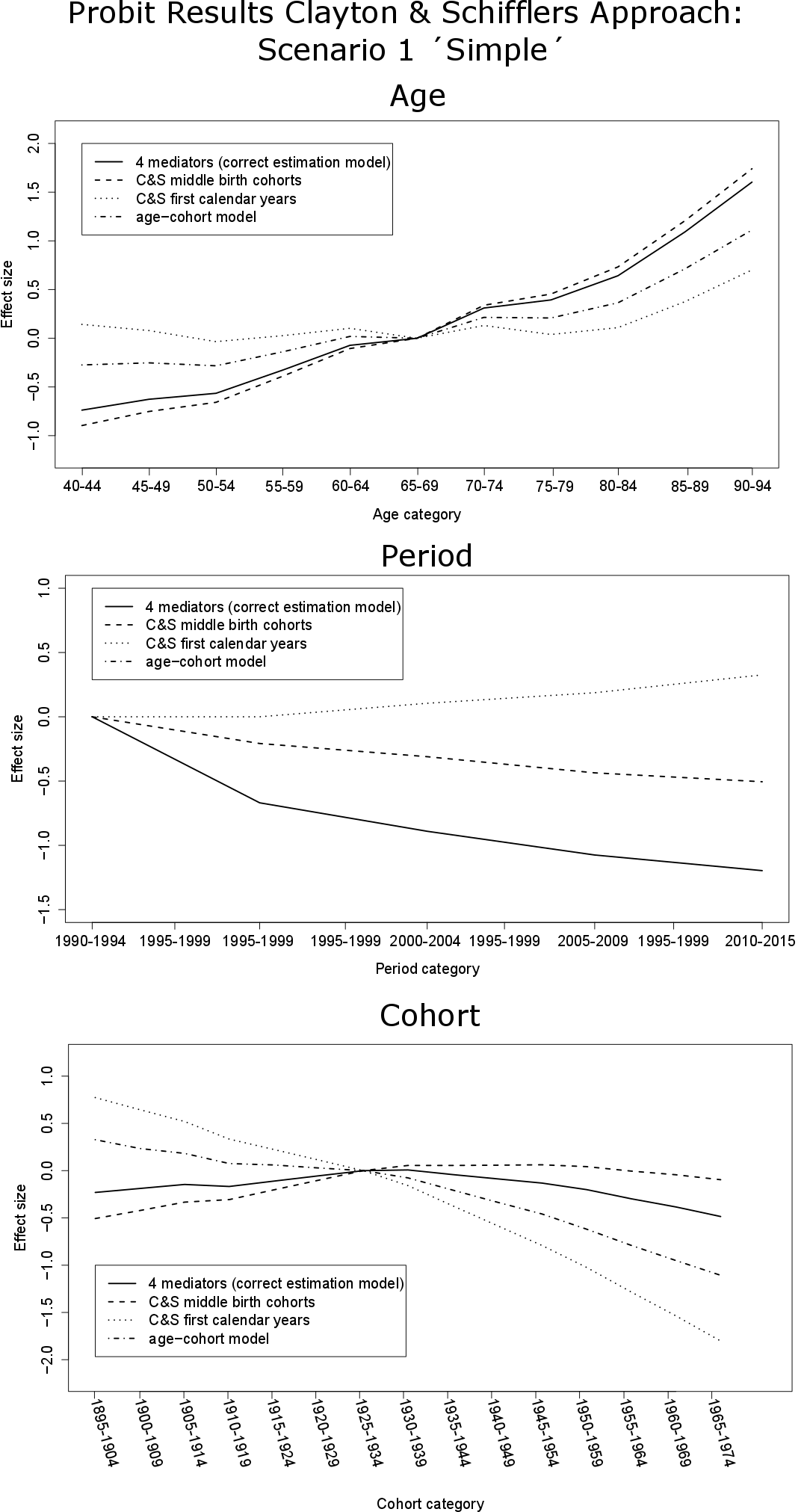


**Figure S9.** Average APC estimates in scenario 1 (simple) using models with 1931-1939 and the 1936-1944 cohort effects constrained to be equal (‘middle birth cohorts’) and 1990-2000 and the 1995-2005 period effects constrained to be equal (‘first calendar years’): summary of 1000 simulations. The correct model specification and an age-cohort model are used as comparisons.

*Comparison with Mechanism-Based approach*

We compare this ‘traditional’ approach with the mechanism-based approach. We used two common types of constraints in our application of the traditional model; one model with constraints for birth cohort and one with constraints for period. Substantively, it is believed that age has the strongest effect on most outcomes, including cardiovascular outcomes, and hence should not be constrained (Hobcraft et al. 1982). In our simulations, constraining two birth cohorts to have equal effects led to better estimates than constraining period categories because in our simulations the birth cohort parameters had similar values. This assumption therefore resulted in little bias in two of the three APC estimates, but the bias would be stronger if age or period were instead constrained. In the second traditional model, we therefore constrained period, and indeed this resulted in greater bias on average. Given the larger slope of the age effects relative to those of period and cohort, constraining age would have resulted in much stronger bias. Importantly, in a real application, it is unknown which effect is largest and which age, period or cohort categories would have similar effects on the outcome. Furthermore, considering that applied researchers commonly have no additional information on the true shape or effect sizes of the age, period and cohort effects, an unfavourable selection of constraints can easily be made.

**References**

Fienberg SE, Mason WM. Identification and estimation of age-period-cohort models in the analysis of discrete archival data. *Sociological Methodology* 1979; 10: 1-67.

Hobcraft, J., Menken, J., Preston, S. Age, Period, and Cohort Effects in Demography: A Review. *Population Index* 1982, 48 (1), 4-43.

Holford TR. The estimation of age, period and cohort effects for vital rates. *Biometrics* 1983; 39:311-324.

Clayton D, Schifflers E. Models for temporal variation in cancer rates. I: Age-period and age-cohort models; II: Age-period-cohort models. *Statistics in Medicine* 1987; 6:449-481.
